# Eating and Exercise

**Published:** 1858-05

**Authors:** 


### EATING AND EXERCISE.

NEVER take a meal under a feeling of exhaustion from exercise.

Never go into a sick room when very weary.

Never exercise violently just before a meal.

The exercise which benefits invalids and infirm people is that which is moderate, and extended in space or time.

One of the best exercises for women who are not very well, is a walk in the streets, or the fields, with a cheerful companion. To find an exercise suitable for women in-doors is very difficult; sewing is too confining, scrubbing the floor too violent: and under the great variety of circumstances under which women are placed in families, we can do nothing more than to lay down a principle, and let each one act in reference to it: that exercise is best which keeps the body in motion, and interests the mind pleasurably.

Eat your meals with an unanxious, unannoyed, and cheerful heart; and consider he, she, or it your worst enemy that interferes in this direction; for passion, anxiety, alarm, mortification, instantly arrest digestion.

No rule as to quantity or quality of food is of universal application, exceptions will be found in the individual. What "agrees" or is "good for" nine individuals, would not be suitable for the tenth. Hence, in reading any general rule as to health or habits of life, it is the dictate of wisdom to enter on its practice with moderation, caution, and a close judicious observation: the necessity of this is strikingly exemplified in dyspepsia, one man being benefited by an alkali, another by an acid—vinegar relieves one, soda or ley another.

